# A Survey of Security in Cloud, Edge, and Fog Computing

**DOI:** 10.3390/s22030927

**Published:** 2022-01-25

**Authors:** Aleksandr Ometov, Oliver Liombe Molua, Mikhail Komarov, Jari Nurmi

**Affiliations:** 1 Electrical Engineering Unit, Faculty of Information Technology and Communication Sciences, Tampere University, 33720 Tampere, Finland; aleksandr.ometov@tuni.fi (A.O.); oliverliombe.molua@tuni.fi (O.L.M.); 2 Laboratory of Cryptographic Methods of Information Security, Faculty of Secure Information Technologies, ITMO University, 191002 St. Petersburg, Russia; 3 Graduate School of Business, National Research University—Higher School of Economics, 101000 Moscow, Russia; mkomarov@hse.ru

**Keywords:** computing, survey, security, privacy, distributed systems, computational offloading

## Abstract

The field of information security and privacy is currently attracting a lot of research interest. Simultaneously, different computing paradigms from Cloud computing to Edge computing are already forming a unique ecosystem with different architectures, storage, and processing capabilities. The heterogeneity of this ecosystem comes with certain limitations, particularly security and privacy challenges. This systematic literature review aims to identify similarities, differences, main attacks, and countermeasures in the various paradigms mentioned. The main determining outcome points out the essential security and privacy threats. The presented results also outline important similarities and differences in Cloud, Edge, and Fog computing paradigms. Finally, the work identified that the heterogeneity of such an ecosystem does have issues and poses a great setback in the deployment of security and privacy mechanisms to counter security attacks and privacy leakages. Different deployment techniques were found in the review studies as ways to mitigate and enhance security and privacy shortcomings.

## 1. Introduction

The goal of having a huge capacity for storage with efficient scalability has recently been the driving force for different enterprises, organizations, and small companies when switching to Cloud, Edge, and Fog paradigms from standalone execution [1]. Significantly, this shift brings numerous challenges along the way. This work mainly focuses on how security in Cloud, Edge, and Fog Computing systems is achieved and users’ privacy protected from attackers. Essentially, the vision is a holistic management style for personal data at the global centers hosting Edge, Fog, and Cloud.

As of today, security and privacy issues have become a major concern when Cloud providers holding large amounts of data and essential applications share them with customers. As a result of these concerns, related topics present major problems in the computing paradigms research field [2]. Currently, the most attention in each computing model is on protecting users’ privacy from unauthorized groups or individuals gaining access and hindering attacks. Moreover, keeping data integrity intact and also maintaining it is a very vital aspect. This research takes an approach to review the security and privacy aspects in Cloud, Edge, and Fog paradigms [3,4,5].

The rapid and ever-increasing need for novel computational offloading strategies is a great challenge when it comes to protecting personal information and other important data [6]. Historically, Cloud customers possess legitimate access to their individual information and data (in other words, users should have the right as to how, when, and to what extent other people can gain access to their personal information) [7]. Importantly, five different features relating to security and privacy aspects are raised in any order: integrity, accountability, confidentiality, availability, and the preservation of privacy [7,8,9].

Recently, there has been a sharp, universal shift from traditional operations in organizations to embracing innovations such as Cloud Computing and other paradigms. These different paradigms have been the subject of many academic studies and reviews from students and researchers. It is both difficult and very challenging for different Information and Communication Technology (ICT) engineers, researchers, and students to generally keep up with the ever-growing pace of new journals, literature, and article reviews. One important area concerning the various paradigms is the security and privacy aspect, which we shall systematically review based on PRISMA guidelines [10].

The rest of the paper is organized as follows. First, Section 2 briefly outlines the explanation of different computing paradigms. Next, Section 3 provides an outlook on the specifics of security and privacy for each paradigm and their similarities. Furthermore, Section 4 provides the major identified challenges and vulnerabilities. Section 5 concludes the discussion.

## 2. Background on Computing Paradigms

Before diving deeper into the main sections of the paper, a general overview of the different mentioned paradigms needs to be provided. For clarity and consistency, each paradigm is carefully discussed concisely. The reason for discussing each of these paradigms is to have an overview that will guide the understanding of the research goal for this paper, which is primarily the information security and privacy aspects for each paradigm.

### 2.1. Cloud-Related Aspects

Historically, the growth and expansion of the infrastructures of many companies have come from evolving technologies and innovations. Cloud computing is seen as a unique solution to provide applications for enterprises [11]. It uses different components such as hardware and software to render services, especially over the Internet. The possibility of accessing various data and applications provided was originally made straightforward by Cloud computing.

Several industrial giants and standardization bodies attempted to define Cloud computing in their understandings and views. The National Institute of Standards and Technology (NIST) is widely considered to provide the most reliable and precise definition for Cloud computing as “a model for enabling ubiquitous, convenient, on-demand network access to a shared pool of configurable computing resources (e.g., networks, servers, storage, applications, and services) that can be rapidly provisioned and released with minimal management effort or service provider interaction” [12].

Five different models particularly characterize Cloud computing: on-demand self-service, broad network access, multi-tenancy and resource pooling, rapid elasticity, and scalability. Generally, more Cloud computing resources can be provided as required by manufacturers and different enterprises while avoiding interactions with humans involving service providers, e.g., database instances, storage space, virtual machines, and many others. Having access to corporate Cloud accounts is essential as it helps corporations to virtualize the various services, Cloud usage, and supply of services as demanded [13].

Simultaneously, there is a need for broad network access, i.e., accessing capabilities via established channels across the network advance the use of heterogeneous thick and thin customer devices such as workstations, tablets, laptops, and mobile phones [14]. This access leads to the resource pooling aspect, i.e., computing resources from the provider are grouped using a particular multi-tenant model used in serving various clients. The unseen and non-virtual resources are carefully allocated and reallocated according to the customer’s needs. Usually, customers do not understand or access the spot-on position or area provided. However, location specification can be established at an advanced state of situation or abstraction followed by various examples of resources such as network bandwidth, processing, memory, and storage [15].

Such a massive heterogeneous environment leads to the scalability aspect [16]. The growth of a client marketplace or business is made possible due to the tremendous ability to create specific Cloud resources, enabling improvement or reducing costs. Sometimes, changes might occur on the user’s need for Cloud computing, which will be immediately responded to by the platform or system.

Finally, the resource use is keenly observed, regulated, and feedback is given to established billing based on usage (e.g., accounts of frequent customers, bandwidth, processing, and storage). The proper reporting of essential services used can be done transparently if the used resources are adequately looked into, controlled and account is given [12].

From the architectural perspective, big, medium, and small enterprises use Cloud computing technology to save or store vital data in the Cloud, enabling them to access this stored information from any part of the world via connecting to the Internet. Service-oriented and event-driven architectures are the main combination that makes up the Cloud computing architecture. The two important parts dividing the Cloud computing architecture are naturally Front End (FE) and Back End (BE) [17].

**Figure 1:**
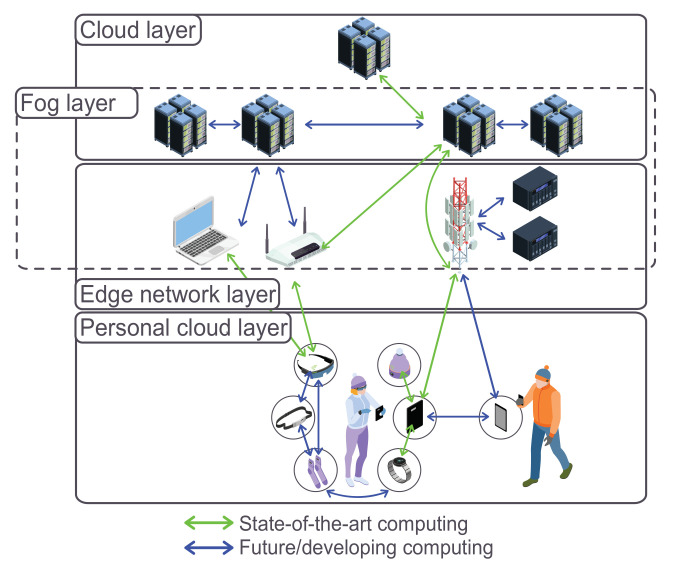
Most common task offloading models.

As seen in Figure 1, various components are involved in the computing architecture [6]. Furthermore, we take a brief look at each architecture’s different features. Furthermore, we can see that a network connects both front and back ends via the wired or wireless medium.

### 2.2. Edge-Related Aspects

As a new generation of computational offloading, Edge arrived to allocate the resources at the network edge, i.e., closer to various office and home appliances such as mobile devices, Internet of Things (IoT) devices, clients, and client’s sensors. In recent years, there has been fast growth in industrial and research investment in Edge computing. The pivot for Edge computing is the physical availability and closeness, of which end-to-end latency is influenced by this essential point of Cloudlets, with bandwidth achievable economically, trust creation, and ability to survive [18].

Communication overheads between a customer and a server site are reduced due to a decrease in actual transmission distances (in terms of geography and number of hops) brought about by the Edge computing in the network. As one of the definitions, “Edge computing is a networking philosophy focused on bringing computing as close to the source of data as possible to reduce latency and bandwidth use. In simpler terms, Edge computing means running fewer processes in the Cloud and moving those processes to local places, such as on a user’s computer, an IoT device, or an Edge server” [19]. Some other definitions of Edge computing are “a physical compute infrastructure positioned on the spectrum between the device and the hyper-scale Cloud, supporting various applications. Edge computing brings processing capabilities closer to the end-user/device/source of data which eliminates the journey to the Cloud data center and reduces latency” [20]. There are several cases in which architectural designs are specifically intended, considering their work plan and setting up the infrastructure is based on its need.

Considered a state-of-the-art paradigm, Edge computing takes services and applications from the Cloud known to be centralized to the nearest sites to the main source and offers computational power to process data. It also provides added links for connecting the Cloud and the end-user devices. One of the best ways to solve or reduce Cloud computing issues is to make sure there is an increase in Edge nodes in a particular location, which will also help in decreasing the number of devices attributed to a sole Cloud [21].

Overall, the main Edge service consumers are resource-constrained devices, e.g., wearables, tracker bands for fitness and medical uses, or smartphones [22]. Fog devices, in turn, subdues the shortcomings of Cloud by transferring some of the core functions of Cloud towards the network Edge while keeping the Cloud-like operation possible [23], e.g., Edge and Fog nodes may act as interfaces attaching these devices to the Cloud [24].

A typical Edge computing architecture comprises three important nodes (see Figure 1): the Cloud, local Edge, and the Edge Device. Notably, Local Edge involves a well-defined structure with several sublayers of different Edge servers with a bottom-up power flow in computation. Both Access Points (APs) and Base Stations (BSs) are Edge servers situated at the sublayer considered to be the lowest together with proximity-based communications [25]. These are particularly installed to obtain data during communication from various Edge devices, returning a control flow using several wireless interfaces.

Cellular BSs transmit the data to the Edge servers found in the (upper) sublayer after receiving data from Edge devices. Here, the upper sublayer is particularly concerned with operating computation work. Very fundamental analysis and computation are done after data are forwarded from BSs. At a recent Edge server, the computational restriction is placed such that if the difficulty in a given work surpasses it, the work is offloaded and sent to the upper sublayers with adequate computation abilities. A chain of flow control is then concluded by these servers with passing back to the access points, and finally, in the end, send them to Edge devices [26].

The Edge architecture allowed to switch more delay intolerant applications closer to the computation demanders, e.g., Augmented/Virtual/Mixed Reality (AR/VR/MR) gaming, cellular offloading, etc., all together following the proximity-driven nature of the paradigm [27]. Generally, there are two approaches to the proximity between the Edge and user’s equipment: physical and logical proximity.

Physical proximity refers to the exact distance between the top segment of data computation and user equipment. Logical proximity refers to the count of hops between the Edge computing segment and the users’ equipment. There are potential occurrences of congestion because of the lengthy route caused by multiple hops, leading to increased latency issues. To avoid queuing that can result in delays, logical proximity needs to limit such events at the back-haul of the computing network systems.

Despite the shortcomings of the normal Cloud paradigm innovations to match up with great demands, given lower energy level, real-time, and in particular security and privacy aspects, the Edge paradigm is not considered a substitute for the Cloud paradigm. Edge and Cloud paradigms are known to assist each other in a cordial manner in several situations. The Cloud and Edge paradigms cooperate in some network areas, including autonomous cars, industrial Internet, as well as smart cities, offices and homes. Importantly, Edge and Cloud paradigm collaboration offers many chances for reduced latency in robust software such as autonomous cars, network assets of companies, and information analysis on the IoT [28].

Nevertheless, Edge operation is executed through supported capabilities from several actors. Cellular LTE, short-range Bluetooth Low Energy (BLE), Zigbee, and Wi-Fi are various technologies that create connectivity by linking endpoint equipment and nodes of the Edge computing layer. There is great importance for access modalities as it establishes the endpoint equipment bandwidth availability, the connection scope, and the various device type assistance rendered [29].

### 2.3. Fog-Related Aspects

Access gateways or set-top-boxes are end devices that can accommodate Fog computing services. The new paradigm infrastructure permits applications to operate nearby to observe activities easily and handle huge data originating from individuals, processes, or items. The creation of automated feedback is a driving value for the Fog computing concept [30]. Customers benefit from Fog and Cloud services, such as storage, computation, application services, and data provision. In general, it is possible to separate Cloud from Fog, which is closer to clients in terms of proximity, mobile assistance for mobility, and dense location sharing [31], while keeping the Cloud functionality in a distributed and transparent for the user manner.

According to NIST, “Fog computing is a layered model for enabling ubiquitous access to a shared continuum of scalable computing resources. The model facilitates the deployment of distributed, latency-aware applications and services, and consists of fog nodes (physical or virtual), residing between smart end-devices and centralized (cloud) services. The fog nodes are context aware and support common data management and communication system. They can be organized in clusters – either vertically (to support isolation), horizontally (to support federation), or relative to fog nodes’ latency-distance to the smart end-devices” [32]. Generally, Fog computing is considered to be an extension or advancement of Cloud computing, as the latter one ideally focuses mostly on a central system for computing, and it occurs on the upper section of the layers, and Fog is responsible for reducing the load at the Edge layer, particularly at the entrance points and for resource-constrained devices [33].

The use of the term “Fog Computing” and “ Edge Computing” refers to the hosting and performing duties from the network end by Fog devices instead of having a centralized Cloud platform. This means putting certain processes, intelligence, and resources to the Cloud’s Edge rather than deriving use and storage in the Cloud. Fog computing is rated as the future huge player when it comes to the Internet of Everything (IoE) [34], and its subgroup of the Internet of Wearable Things (IoWT) [35].

Communication, storage, control, decision-making, and computing close to the Edge of the network are specially chosen by Fog architecture. Here, the executions and data storage are executed to solve the shortcomings of the current infrastructure to access critical missions and use cases, e.g., the data density. OpenFog consortium defines Fog computing as “a horizontal, system-level architecture that distributes computing, storage, control, and networking functions closer to the users along a Cloud-to-thing continuum” [36]. Another definition explains Fog as “an alternative to Cloud computing that puts a substantial amount of storage, communication, control, configuration, measurement, and management at the Edge of a network, rather than establishing channels for the centralized Cloud storage and use, which extends the traditional Cloud computing paradigm to the network Edge” [37].

The deployment of Fog computing systems is somewhat similar to Edge but dedicated to applications that require higher processing power while still being closer to the user. This explains why devices belonging to the Fog are heterogeneous, raising the question of the ability of Fog computing to overcome the newly created adversaries of managing resources and problem-solving in this heterogeneous setup. Therefore, investigation of related areas such as simulations, resource management, deployment matters, services, and fault tolerance are very simple requirements [38].

As of today, Fog computing architecture lacks standardization, and until recently, there is no definite architecture with given criteria. Despite so, many research articles and journals have managed to develop their versions of Fog computing architecture. In this section, an attempted explanation is detailed in an understanding manner, which describes the different components which make up the general architecture [38].

**Figure 2:**
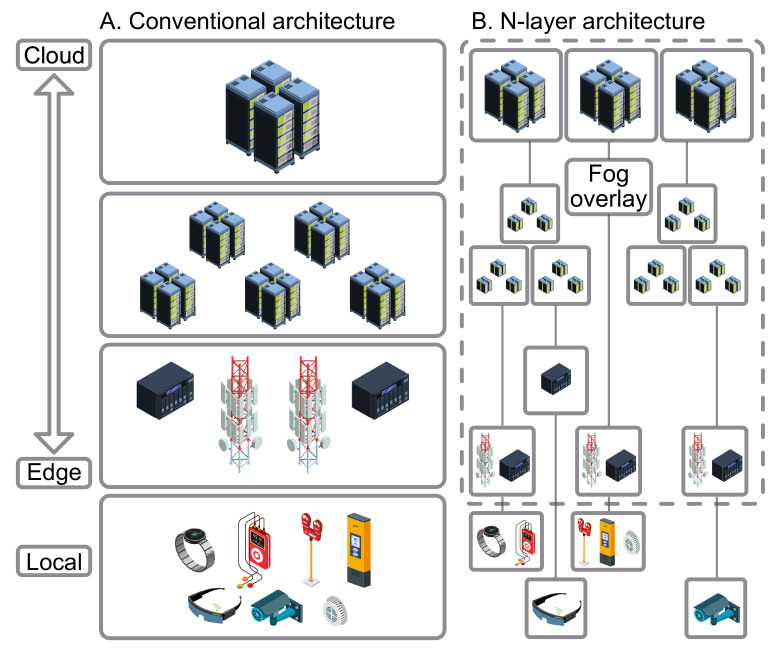
Most commonly analyzed computing architectures.

Generally, most of the research projects performed on Fog computing have mostly been represented as a three-layer model in its architecture [39], see Figure 2. Moreover, there is a detailed N-layer reference architecture [40], established by the OpenFog Consortium, being regarded as an improvement to the three-layer model. However, we will be looking at a three-layer architecture.

Fog computing is considered to be non-trivial addition regarding Cloud computing based on Cloud-to-Things setup. In fact, it displays a middle layer (also known as the Fog layer), closing the gap between the local end devices and Cloud infrastructure [41].

Notably, and as in the Cloud, the Fog layer also uses local virtualization technologies. On the other hand, taking into consideration the available resources, it will be more adequate to implement virtualization with container-based solutions [38]. It should also be remembered that Fog nodes found in this layer are large in number. Based on OpenFog Consortium, Fog node is referred to as “the physical and logical network element that implements Fog computing services” [42]. Fog nodes have the capability of performing computation, transmission, and also storing data temporarily and are located in between the Cloud and end-user devices [43].

The essential pushes for the eminent migration from Cloud computing to Fog computing are caused by load from computations and bringing Cloud computing close to Edge. Several characteristics define Fog computing by the tremendous variety of applications and IoT design services [44]. The major one corresponds to the extreme heterogeneity of the ecosystem, which provides services between centralized Cloud and different devices found at the Edge, such as end-user applications via Fog. The heterogeneity of Fog computing servers comprises shared locations with hierarchically structured blocks.

At the same time, the entire system is highly distributed geographically. Fog computing models consist of extensively shared deployments in actuality to offer a Quality of Service (QoS) regarding mobile and non-mobile user appliances [45]. The nodes and sensors of the Fog computing are geographically shared in the case of various stage environments, for instance, monitoring different aspects such as chemical vats, healthcare systems, sensors, and the climate.

The ability to effectively react to the primary goal and objective can be called cognition. Customers’ requirements are better alerted by analytics in a Fog-focused data gateway, which helps give a good position to understand where to make a transmission, storage possibilities, and the control operations along the whole process from Cloud to the Internet of Things continuum. Customers enjoy the best experience due to applications’ closeness to user devices and creating a better precision and reactiveness concerning the clients’ needs [46].

### 2.4. Differences and Similarities of Paradigms

The main goal of Fog and Edge paradigms are similar in some areas, unlike the Cloud. Both of those bring the capabilities of the Cloud closer to the users and offer customers with lower latency services while making sure, on the one hand, that highly delay-tolerant applications would achieve the required QoS, and, on the other hand, lowering the overall network load [47]. It is not straightforward to differentiate and compare Cloud, Edge, and Fog Computing. This subsection attempts to discern and look into similar features between the computing paradigms [48]. The differences and similarities of the various paradigms are summarized in Table 1.

Nonetheless, it is essential to overview each of these indicated paradigms to address security and privacy aspects in Cloud, Edge, and Fog paradigms. This subsection described some fundamental features that constitute each of the said paradigms, making them unique in their ways. We looked into the different architectures, how these paradigms are characterized and how beneficial they are to the industries, and addressed some scenarios in which they are applied.

Cloud being a centralized architecture and an IoT promoter has several shortcomings such as high latency, location sensibility, and computation time, just to name a few. Researchers then suggested upgraded technologies known as Edge and Fog paradigms to lessen the burden on Cloud systems and resolve the issues indicated. Ultimately, we see that those two paradigms have helped decrease the large quantity of data sent to the Cloud.

Finally, the Edge paradigm is advantageous over the Cloud paradigm, especially regarding security and privacy. However, the Fog paradigm consisting of Fog nodes is regarded as an outstanding architecture uniquely created so that IoT appliances render improved services and support. Next, we shall present some security and privacy analyses relating to Cloud, Edge, and Fog paradigms, respectively.

## 3. Security and Privacy of Computing Paradigms

Security and privacy have a symbiotic relationship and are closely related. Many academics and organizations see the two terms closely related to the ICT domain. The influence of digitalization has tremendously shaped our daily activities [30]. Industrial giants currently deal with various computing paradigms involving huge computation and processing of Big Data. Thus, transmitting these data from one source to another makes it vulnerable and requires protection. In this section, we will define security, privacy, threats, countermeasures, and security mechanisms, and we will see some differences and possible similarities between security and privacy [49].

### 3.1. Cloud-Related Aspects

The majority of today’s networks and the idea of storing data remotely is greatly inclined to technologies relating to Cloud computing. One of the exceptional demands is for the Cloud to see that services are always made available consistently, the reliability is maintained, and data are supplied as demanded. As mentioned earlier, one of the prime reasons organizations or individuals are reluctant to embrace the quick movement to the Cloud model is the huge concern for information security and privacy. Some acknowledged issues tied to security and privacy in Cloud computing include confidentiality, data security, phishing, and multi-tenancy [50]. This section looks into the various threats aligned with security and privacy within the Cloud computing system and suggests some modalities for threat mitigation.

Cloud computing users adopt different distributed Cloud models based on their specific needs, and because of this, the Cloud security and privacy threats differ according to the infrastructure hosted in the Cloud. According to the Cloud Security Alliance (CSA), major regular threats are information leakages, Denial of Service (DoS) Attack, and Advanced Persistent Threats (APT) [51].

Adequate Cloud infrastructural security largely depends on the established protective technologies with many layers. This brings about the importance of adapting an Intrusion Detection System (IDS) specifically to trace suspected threats intelligently and intercept potential attacks over a network. Furthermore, the various events witnessed can be separated to carry out network status analysis. Resources and services of Cloud CIA are said to encounter different types of threats originating from either inside or outside intruders [52].

#### 3.1.1. Cloud Data Security

Data security is an essential aspect that plays a significant role in handling Cloud devices and keeps them running. This may involve protection and restoration guides for data and centers for Cloud services, and data involved in transmissions or transfers must always be protected.

Generally, there is a need for simple yet robust mechanisms that offer a smooth method of learning about Cloud service capabilities before deployment and those that align with Cloud security features during the establishing stage. The presence of Cloud service providers and Cloud customers also plays a role in the deployment plan since both parties must meet certain data security requirements [53]. Here, issues such as service level negotiation, information traffic, and especially data security will arise [54]. It is important for Cloud service suppliers to properly protect customers’ data stored in the Cloud to reduce or eliminate security shortcomings. Techniques used in encrypting data must be very strong to guarantee better data security and implement authentication mechanisms that monitor other information access. Access control through data encryption should be established so that only the rightfully selected employees can reach the data.

#### 3.1.2. Cloud Data Privacy

The public Cloud faces more privacy threats, although these threats are very different based on their Cloud model variants. Some of the concerns of the danger here are the proliferation of information, malicious usage by an unauthorized person, and incapability to control by clients [55]. Clients’ sensitive documents stored in the Cloud can be reached by attackers using the file’s hash codes, with the help of a mechanism used in duplicating information [56]. Risks about privacy are regarded from several angles, such as access control, Cloud systems, customers, and stored information [57]. Knowing data privacy and other relating privacy principles will enormously assist in dealing with the known threat concerns. One vital setback holding some organizations from moving to the Cloud is the fear of losing classified data through information leakage [58].

Most often, people’s privacy is breached either knowingly or unknowingly. Accessing a person’s private data without their knowledge or authorization is strongly considered an invasion of privacy. Different trends can occur, such as open disclosure, privacy attack, data violation, and other means of attacks. Privacy leakage can be very damaging, but privacy issues can be better managed with the points mentioned below:

- Trust: Disclosing data of an individual or organization is considered a breach of privacy. Trust plays a very pivotal role in decreasing or eliminating fear [59]. There are various trust standards every customer can agree to, but in general, their concern is to see minimal or zero breaches of privacy at a reasonable scale [60].
- Access Control: Cloud systems present massive issues, such that an unauthorized person or group of individuals can obtain access if not properly addressed. An effective way of handling this is by answering the questions [61]:
  - Who? The privileged persons to access certain data and who not to.
  - What? Some detailed data are not made accessible to every worker. So what specific files are permitted for whom?
  - When? Some data are needed for a period of time, and that period must strictly be controlled when that information has been accessed.
  These can be made functional by establishing management policies, checks on multi-domain, and providing strong management keys.
- Encryption of data needs to be sufficiently strong to protect the privacy of the client’s files. Weak encryption of data poses a serious challenge to Cloud privacy [61].

### 3.2. Edge-Related Aspects

Since Cloud computing’s performance dropped greatly caused by various factors, including the growing number of nodes, Edge computing has provided a significant paradigm shift. Edge Computing is observed as an innovation because it can carry applications with its new technological capabilities in shared computing while also performing information processing right at the point of need, without transporting the data to the Cloud. Users overall have a better feeling when data are processed close to them, improving their response time. This is made possible thanks to the computation that is directly carried out at the nodes of distributed equipment [62].

Fifth Generation (5G) networks are taking over many areas and operations of our daily activities [63]. Edge computing is undeniably the pivot of all these changes being a part of 5G network, making it vital in terms of smaller resource-constrained devices and how they interact. Edge Computing shows a relationship with heterogeneous equipment and several cross-connected networks. The inter-connectivity of these Edge supporting technologies exposes it to the most concerning aspect of any device, technology, network, and above all, organizations, which is safety. The threats involved here cannot be taken for granted, and this now led us to the subject matter, security, and privacy in Edge Computing. With computation at the node of Edge devices, other security circumstances will show up and still require continuous research work for improvements [64].

In Edge, the chances for imminent threats and attacks are very likely because of the decentralized design of the Edge computing system, even though the processing of information at the nodes offers some security and privacy protection. Smart devices also expose security issues and dangerous malware to Edge computing. The structure of Edge computing cannot adequately support the mechanisms for securing and protecting information. This, therefore, implies that the complexity of this Edge node at the network leaves the data very exposed and hard to secure.

Despite the growing nature of Edge computing technologies, its security and privacy development remain a continuous process and tells why there exist not so many research findings. Researchers and other academics globally have been putting every effort in performing relevant research work to develop countermeasures to improve the security and privacy of Edge systems. Different simple mobile Edge computing methods were used for carrying out security checks, presentation of an overall security and protection scheme with proposals from the research work done. The Edge security findings do present a relevant citation from a theoretical approach. As mentioned previously, the existing known issues in this work relating to Edge computing information security and privacy are partitioned into four separate parts [65]: Access Control, Identity Authentication, Information Security, and Privacy Protection. Based on the focused theme of this work, “Security and Privacy Aspects”, we shall be looking more into only Information security and data protection.

#### 3.2.1. Edge Data Security

Data integrity, confidentiality, and attack detection are the common goal and reasons for data security. It assists in designing an Edge-computing system that is secured. Issues such as information breach and information loss are resolved by outsourcing information under control, non-fixed storage, and sharing responsibility. Data duties are allowed to be carried out securely by customers. Presently, it is still challenging to identify works on Edge Computing security, and privacy since many academics do mostly focus on Cloud paradigms [66], or perhaps Fog paradigm [67]. The major aim of information security in Edge systems is to securely move data and ease the heavy load by creating a shared model with a smoothly operating system. As a result, very acceptable shared information security and lightweight designs are developed for both end-users and remote nodes.

A key responsibility in safeguarding customers’ secrets and upholding the confidence involved, especially at the Edge network, should be rendered, e.g., a digitalized building constructed with many IoT devices, which can be a prime target due to its huge quantity of personal data produced. Therefore, a more regarded approach to protect the privacy of customers and gain their confidence is to make sure that data processing occurs at the Edge network or node of the house [68].

In addition to aspects detected earlier, the following notable Edge-specific elements should be considered. Note, cloud challenges also generally apply to Edge operation scenarios:

- Confidentiality, in the case of mobile clients intending to use the services of mobile applications, is always taken seriously, and for this reason, some clients find it difficult to decide whether to use it [69]. The authors of [70] list some shortcomings relating to Edge computing confidentiality, showing a very high risk posed by the providers of services gaining unpermitted passage to classified information. This occurs during data transmission in a distributed or unsecured network later stored and processed in the Edge distributed network. Data security has constantly been breached. Good enough, restricting access today to project confidentiality is achievable due to some newly created mechanisms [71].
- Detecting Attacks: Edge systems can operate smoothly with the assistance of Edge nodes where the Edge applications are located to offer maximum standard services. This ensures that the entire Edge system is free from abnormalities or threats. The Edge node consists of harsh surroundings with an inadequate security guarantee, exposing the Edge nodes to threats. The performance of an Edge system can massively be hindered when the threats from one Edge node are mismanaged and might subsequently extend to another Edge node. Thus, finding a quick solution can be hard because of the weight of the threat that spreads across the Edge nodes. Furthermore, added costs would be incurred to find the baseline reason for the problem, and even recovery might take a while [72]. Therefore, regular checks must be performed to detect any previous potential or imminent attacks.

#### 3.2.2. Edge Data Privacy

In Edge computing, accessing the system does not reflect trust. Averagely accepted systems store important data, resulting in critical privacy leakage. Examples of clients’ data stored are personal information, location, and identity. The focus areas to be discussed herein any order include privacy, identity, and location privacy safeguarding [73].

Edge computing always raises much concern in stark contrast to other existing computing models protecting information. This is because the challenges, e.g., leakages relating to Edge data privacy, are daunting. An Edge information center, services, infrastructure suppliers, and even certain clients are the potential weak link or at least establishments you cannot fully trust with such interwoven computing/cellular networks. With regard to this, the act of keeping safe the private information of clients is an obligation that requires very close attention [74]:

- Protection of Data Privacy: At the Edge nodes, huge amounts of data belonging to clients are retrieved from applications and other users’ pieces of equipment. This collected information is then processed and analyzed. Despite the trustworthiness of the Edge computing nodes, they can still display some level of vulnerability. Classified information such as an individual’s medical data must be top secret. Therefore, information privacy protection is very important to avoid leakage at the nodes of Edge computing [75].
- Identity Privacy: Compared to the Cloud systems, especially Mobile Cloud, Edge models still lack adequate research attention in protecting the identity of customers well. Identity privacy protection is a major concern for several organizations and even individual customers. The third-party identity-designed model is said to still pose vulnerability [76].
- Location Privacy: Several software and services from Worldwide Web render functional capabilities based on location. For a client to gain access when they want to use the services in Edge computing, that client must deliver their location as required by the service provider [77,78]. One of the particularly concerning fears is breaching data location through possible leaks. Different researchers gave some solution schemes on how to deal with issues on data leakage. A dynamic distribution in location privacy protection was presented in a mobile model of social internet platforms. This model can sort out visitors with low trust levels within a certain range of social interactions. It performs this by dividing customers’ data location (unidentifiable) and personalities in individual storage systems. This separation enables the service provider to hide customers’ location data safely. The importance of this model is that even if an attacker manages to breach one of the storage facilities, for example, data location, it will not pose a major threat since the identity of the client is not leaked or exposed [79].

### 3.3. Fog-Related Aspects

Many businesses have transformed massively, especially with the fast growth in large data usage, due to Cloud computing [80]. Meanwhile, the quest for private services also began to grow hugely. A great number of well-centralized systems is offered by Cloud computing platforms [81,82], although with some shortcomings. Clouds and their endpoints show certain unwanted long and irregular delays and time-conscious services to some [83]. There is a pertinent high risk in a situation whereby there is a breakdown in the information building and between network interconnected systems. One potential breach here is possible privacy exposure. To mitigate this challenge, the Fog computing [84] model was introduced, and it assisted Cloud-Edge in improving computation, security, and privacy, which is now the leading and most recommended computing service.

Fog devices are considered to be separate and distributed pieces of equipment ranging from gateways, routers, switches, or professional installation of traditional servers [85]. Furthermore, with the current demand for huge emission reduction, Fog computing is highly viewed as a smart green platform with sustainability and great security benefits. Many fog Nodes (FNs) are seen as renewable constitute the Fog computing system. The geographical placing of FNs can be spread throughout several locations. A great level of pressure exerted in the information center during computation is vastly decreased due to the different FNs working independently but together through a well-calculated formula. Fog can separate or sifter the processing at the central layer found at the middle of the endpoint and Cloud [86], which may significantly enhance the QoS and brings down expenses [87]. Fog computing was highly considered in great demand to deal with the ever-growing IoT issues, as we shall see in the next sub-Section [88].

Fog computing was established as the most viable approach because of its ability to cross-connect every digital equipment, wireless endpoint, and local device. This interconnectivity is vulnerable to vital security and privacy violations such as disclosing clients’ data location, leaking classified documents, and stealing private accounts. First considered by Cisco, Fog computing was brought to expand the Cloud activities to the system’s Edge. The consideration of Fog computing surfaces as an option to local Cloud offering huge assistance in terms of QoS, latency, and location distribution [45]. Services such as networking, storage, and most importantly, computing between the customer and information center are rendered by Fog computing hugely considered a virtualized system [89], carrying the related vulnerabilities along the way.

According to the Edge system, every single unit in the Edge computing functions independently to see that information is not forwarded to the Cloud, and instead, it is locally handled. On the other hand, transferring to Cloud or processing the data from various information origins is always a decision made by Fog computing nodes, taking into account its assets. Fog computing can expand some Cloud services that are not assisted in Edge structure, such as Infrastructure as a service (IaaS), software as a service (SaaS), and platform as a service (PaaS). Fog computing is completely Edge inclined but can be supported by Fog computing while at the Edge of the network, expansion of communication assets and computation are performed [90].

#### 3.3.1. Fog Data Security

Some attacks usually threaten private and government entities since they function in Cloud, Edge, and Fog computing. To offer a level of protection to the architecture, a Threat Intelligence Platform (TIP) is important to be developed [91]. Data security is the most prioritized aspect in the industrial sector, especially as information must be safeguarded. Intelligent equipment and sensor devices are deployed to reduce threats and security attacks extensively. The feature about heterogeneity and geographical sharing impacts the implementation of Cloud security frameworks into Fog computing systems [5]. Some of the considered security challenges are confidentiality, authentication, availability, and information privacy. These mentioned frameworks assist in creating and monitoring accesses to persons and organizations.

Considering the medical field, we see that patients’ health history involves classified information and the Fog architecture has several nodes that might present some vulnerabilities. These vulnerabilities can be unpermitted access to information when stored or at the time of transfer, untrustworthy insiders, and during system distribution of information. Fog system by means of cable or wireless network consistently receives information transferred from sensors of medical devices. Tampering with patients’ personal data, integrity, and device availability is obvious and can occur when communication systems and sensors are targeted. Some through channels as Denial of Service (DoS) can easily be perpetrated due to the vulnerabilities found in wireless networks. On the other hand, the absence of proper frameworks to control access to the Fog nodes that process important information can compromise information through leakage because of account theft, unpermitted access, and possibly some unsafe passage. The mentioned problems can be mitigated through thorough analysis and stringent rules and regulations to establish standard control mechanisms such as personal systems, selective (limited) encryption, and reciprocated authentication [92].

Overall, Fog provides Edge-like challenges while bridging those even more towards the decentralized and distributed environment.

#### 3.3.2. Fog Data Privacy

Protecting the privacy of individuals and enterprises is often a primary concern encountered by the Fog paradigm, especially with the Fog nodes positioned near the individuals and facilitates the gathering of vital information sometimes relating to geographical location, identity, social security numbers, and many. One great challenge is that it is quite hard to keep centralized monitoring due to the distributed nature of Fog nodes.

During transmission, attackers can easily gain access to steal essential information when the Fog nodes are not well secured. More practical studies are needed to understand privacy problems better and innovate current solutions to preserve data privacy [93]. Privacy leakage often happens, even though end-users are never in accordance to release their personal information. There are some main areas of clients’ privacy: data privacy, location privacy, identity privacy, and usage privacy [94].

## 4. Main Security and Privacy Challenges

This section briefly describes the major challenges per paradigm and provides a concise table highlighting the essential ones and the proposed countermeasures identified in the literature.

### 4.1. Cloud Paradigm Challenges

Data loss, privacy leakage, multi-tenancy, unpermitted access to management platforms, Internet protocol, injection attacks are some of the main challenges faced in Cloud [95,96]. Such challenges turn to make room for potential attacks, letting access control to cybercriminals, granting access to unauthorized services, therefore disclosing several classified data, if not all.

Cloud computing faces enormous threats when involved with these vulnerabilities and thus affects business too, either directly or indirectly. One of the most reliable ways to repel threats and attacks is to identify any found and analyze the behavior properly. This section explains the different Cloud computing issues [97].

- Multi-tenancy is used in providing services to different customers and organizations with a particular software operating on the SaaS provider’s servers within the architectural design. Every user company can use an application that is virtually designed in dividing data and configuring it virtually with the help of specially designed software. In this SaaS model, there is a high risk of vulnerability because clients turn to work with applications of multi-tenancy manufactured by Cloud Service Providers (CSP). The maximum-security of customer’s data is the direct responsibility of the Cloud provider since sensitive information such as financial and individual data are hosted in their Cloud system [55].
  Managing resources and scheduling work are some methods used by certain Cloud providers [98], but hardware potential is fully attained through virtualization by CSPs providers. Sandboxed setups refer to Virtual Machines (VM)being completely separate. Hardware sharing with the clients is considered safe according to this mindset. On the other hand, cybercriminals can gain access to the host when the sandboxed system has security setbacks [99]. The virtualization software is strongly recommended since it is capable of showing recent vulnerabilities in Cloud security, such as retrieving data by targeting a VM on one machine through attacks through cross-Virtual Machine side channel [100].
- Data Integrity: Security attention is greatly put on data integrity in the Cloud, which means any reply to a data request sent must be from someone with an access privilege. Establishing a general basic data integrity standard is important, though it is not still in place [101]. Trust is one of those many values that clients are expected to demonstrate in the computing facet. Today, a lot of companies or institutions encounter the issue of trust, and this hugely impacts the handling of their data [102].
- Unauthorized Access: One of the most vulnerable aspects of Cloud computing is giving unauthorized access to management platforms and resources. Users are exposed to this due to the shared technologies often involved in Cloud services. An acceptable way of mitigating the security solution of such a scenario is by introducing access control, and this helps in securing the client’s personal information and its domain for privacy [103]. It is worth noting that cybercriminals can simply have unauthorized access to Cloud service systems because of a single-style authentication model and not very strong authentication mechanisms being used [104].
- Data loss and Leakage: The low cost of Cloud services is one reason customers turn to migrate to the Cloud, and it is warned that customers should pay attention to their important information since various diverse aspects can easily breach their data security. There is an increased chance of data leakage or loss due to high traffic and usage of the Cloud. The vulnerabilities and threats in Cloud service are undeniable, posing a great security threat to businesses and institutions. Significantly, it can be frustrating when you cannot retrieve and restore data after accidentally deleting files from the Cloud due to a lack of a backup system [105].
- Malicious Insider: Every organization has different rules and regulations regarding recruitment policies and employee information. However, some employees have higher status, which guarantees them the privilege of accessing certain essential data within the company. Based on CSA, they proposed the implementation of transparency in the general data security and management activities standard, outlining notification procedures during security failures, while using Service Level Agreement (SLA) as a demand for human resource, and finally establishing and exercising strict rules in the management of supply chain [105].
  It may be far easier for a person with malicious ideas to work for a CSP since no one is seen as a suspect [106]. This individual can quickly be involved in malicious events, especially if they have unhindered access to sensitive information, especially if the CSP cannot strictly monitor its workers.
- Identity Theft: Victims or organizations can suffer heavy impact due to weak passwords due to phishing attacks by some attackers who turn to disguise as authentic persons to steal the different important data of their victims. The sole reason for identity theft is to gain access to sensitive digital resources of individuals and companies by any malicious means. Every protected communication within the Cloud system happens with access control, and this is made possible using an encryption key [107].
- Man-in-the-Middle Attack: During the flow of data from one end to another or between different systems, cybercriminals can easily take advantage and gain access, therefore having control of classified data. This can easily occur when the secure socket layer (SSL) is insecure due to inadequate configuration. Specifically, in Cloud systems, hackers can attack the communication within the information centers. Efficient SSL configuration and data analysis among accepted entities can go a long way to significantly lower the threat posed by a middle-man attacker [108].
- The DoS attack aims to limit or stop the execution of service and from accessing needed data. This creates a scenario where actual users partially or fully lack service availability. Whenever the right person uses the Cloud services to reach the data server to access information, access is denied. This happens because the attacker uses a method in which he constantly congests the server of a precise resource through request flooding, and the targeted server will then be unable to reply to a legitimate access request. There exist several ways this attack can be performed, for example, by way of SQL injection attack, bandwidth wastage, and also by way of incorrectly using model resources [109].
- Phishing Attack is one of the most common attacks in which the criminal turns to impersonate and deceive their victims by leading them to malicious links. The presence of the Cloud makes it flexible for hackers to hide their Cloud hosting of numerous accounts of different clients that uses Cloud services using phishing activities. There are two kinds of threat divisions in which phishing can be grouped. Primary, irresponsible attitude whereby a cybercriminal can also make full use of Cloud services to simply host a site for a phishing attack. Secondary, Cloud computing services and their many accounts can be hijacked [110].

### 4.2. Edge Paradigm Challenges

The Edge paradigm is considered to offer huge benefits to Edge customers such as storage, data processing, just to name a few. However, unlike the Cloud paradigm, Edge computing still faces big security and privacy challenges, which we will explore despite these many gains in this subsection.

- Data Injection: When a machine is vulnerable, an attacker can push harmful information to share negative information. The act of injecting dangerous data by a malicious attacker into a device is known as poisoning. Data can be faked, then used to create fraudulent messages to render the nodes of the target compromised, and it is called an external forgery, for example, in a modern digital industrial production line where the adversary happens to give false machine readings, therefore causing severe functional changes with the bad aim to harm the devices [65].
- Eavesdropping: In this scenario, an attacker can mask itself and observe network traffic during transmission and capture data illegally. It is quite hard to point out this type of attack because the attacker happens to hide inside the platform [111].
- Privacy Leakage: The absence of strict access control to the node of Edge can easily lead to data privacy being tampered with. However, the attack strength is very low. The information generated from devices situated at Edge proximity is stored and processed in the Edge data building. Customers classified these Edge data buildings can leak information since the content is known [112].
- Distributed DoS: Attackers usually take advantage of network protocol vulnerabilities to launch attacks on Edge nodes, causing network damage and restricting resource access and provision of services. Attackers carry out these attacks by loading the server with many data packets to shut down the channel by jamming the server’s bandwidth. Another option is where the Cloud data server or the Edge systems are being flooded with data packets to massively take out resources [65].
- Permission and Access Control: Unauthorized access is a major challenge in the Edge paradigm. It is important to know an individual or employee before authorizing them to access any sensitive information in the system. It can be achieved by establishing access control protocols. Connectivity between several pieces of equipment and other services can be considered secured when access control measures and permission are implemented [113].

### 4.3. Fog Paradigm Challenges

The Cloud paradigm has countermeasures for its security and privacy threats. Nevertheless, these countermeasures may not apply to the Fog paradigm due to the active presence at the network Edge of Fog entities. The immediate vicinity where Fog entities operate will confront various threats which may not constitute a good functioning Cloud. The security solutions in the Fog paradigm are improving and increasing as well. However, most of the published literature on Fog computing security and privacy does not provide insights with an extensive assessment of the various issues. Importantly, we elaborate on some security and privacy challenges encountered in the Fog paradigm.

- Trust Issue: Fog systems face trust design challenges due to the reciprocal demand for trust and the distributed nature of their network. Cloud computing platforms are different since they already consist of pre-designed security models that match the industrial security requirements, granting customers and enterprises some trust measures within the Cloud system. However, this is not so with Fog computing networks which are more exposed and liable to security and privacy attacks. Even though the same security mechanism can be deployed to every Fog node that makes up the Fog computing network, the distributed design also makes it quite challenging to resolve the trust problem [24].
- Malware Attacks: Infecting the Fog computing system with a malware attack is a very high-level challenge in the network. It is carried out to steal sensitive data, breach confidential information, and even refuse service with the help of a virus, spyware, Trojan horse, or Ransomware. To assist Fog computing applications in mitigating these malicious attacks, authentic defense mechanisms for virus or worm detection and advanced anti-malware must be introduced [114].
- Computation—Data Processing: Fog nodes often receive data collected from end-user equipment, processed, sent to the Cloud system, or end-user pieces of equipment are forwarded information transmitted from the Cloud. After the various processes, the data sent from end-users to Cloud systems and the data sent from Fog nodes to the Cloud are different in size and nature. Another challenge here is that several providers have these Fog nodes, making them hard to be trusted due to the many security and privacy shortcomings arising after the processing of data [115].
- Node Attack: Here, the attacker engages physically by targeting to capture the vulnerable nodes. There are moments when the attacker can decide to alter the whole node, cause defects to the hardware, or steal sensitive information from the Fog nodes by digitally sending messages and causing sensor nodes distortion of classified data. Such attacks can have damaging effects on the nodes of the Fog network, and observing these node sensors will help identify issues and deploy some node capturing defense of algorithmic cryptography [114].
- Privacy Preservation: There is a huge concern as customers using CSP, IoT, and wireless systems face data leaks of personal information. It is not easy to preserve this privacy in the Fog network due to the closeness of Fog nodes to the customers’ environment, and it can also facilitate gathering plenty of vital information such as identity, location, and utility usages. Privacy leakage can also occur when communication between Fog nodes becomes more frequent [94].

### 4.4. Major Attacks and Countermeasures

It is essential to note that vulnerabilities, threats, or security attacks can appear differently in different paradigms, and there exists no specific way of solving the various security issues. Thus, several designed models must be considered to safeguard a Cloud, Edge, or Fog computing system. This will help create a joint force of many reliable layer defense models [116].

Table 2 presents a detailed comparison of Cloud, Edge, and Fog paradigms based on a designated OSI model layer. Different attack examples were common to the three involved paradigms associated with the various layers. These identified security attacks and privacy leakages are matched to a specific proposed countermeasure. In some situations, the same countermeasure of a particular paradigm can be applied to the other ones. However, due to the complexity of these paradigms or their ecosystem, this deployment of a single countermeasure is challenging.

As of now, end devices do not involve any established security measures. For this reason, during data transmission, security vulnerabilities are likely to be present. Some vulnerability research is underway to understand the different ways an end device or layer can face an attack. It is of significance that vulnerability research projects must be carried out extensively and in-depth when studying attacks and their aspects [141]. At each layer, we can deduce that security vulnerabilities are safeguarded differently. This attains the basic security demands such as confidentiality, authenticity, integrity, and not the least, availability. Cryptography is suggested for data confidentiality in stopping data leakages to illegitimate persons. Although cryptography turns out to offer better data confidentiality, it does need additional computation power, therefore causing latency. Users and end-devices have proximity to each other. For example, FNs pose some level of reach to individuals’ data, especially where the information is generated. Data processed in FNs are significant security-wise due to their sensitivity more than data being processed in Cloud servers, thus requiring enhanced protection.

Overall, Cloud, Edge, and Fog paradigms consist of applications, resources, and a massive quantity of end-devices within a given centralized or decentralized area, existing together and inter-communicating. Therefore, the huge potential for vulnerabilities in security and privacy does exist. One good way of screening systems for possible vulnerabilities is by auditing security standards.

Vulnerabilities in any system might expressly grant attackers partial or full access to cause severe harm. If data are breached, it can expose critical information of individuals or organizations, and an attack can cause serious malfunctioning of an entire network and create disruptions. We found that the main target of gaining access to sensitive data is threats, seizures, or vulnerabilities of the examined paradigms, whether joint or apart.

Importantly, we found that these vulnerabilities can be properly discovered with the right tools and approaches. Despite the constant search for vulnerabilities in systems by attackers (hackers/cybercriminals), there are up-to-date, sophisticated countermeasures to mitigate such threats, internal or external. Most essentially, each vulnerability has a specific mechanism to counter its threats and attacks. Moreover, another important aspect is that the vulnerabilities turn to undermine the security and privacy of the related paradigms, exposing them (data) to potential security attacks and privacy leakages.

## 5. Discussion and Conclusions

The essential aim of this work was to execute a comprehensive article review on Cloud, Edge, and Fog paradigms, respectively, with a special focus on identifying similarities, differences, attacks, and countermeasures based on security and privacy aspects.

Cloud, Edge, and Fog paradigms create a substantial heterogeneous quantity of data capable of being managed over a centralized or distributed system. Looking at the discussions presented in this work, we deduced that the security and privacy issues on the heterogeneity of this ecosystem are a significant challenge. Data transfer from one end to another opens a way for many security and privacy vulnerabilities, even though some of these weaknesses can be detected and eliminated quickly. Solutions cannot be swiftly deployed to user devices simply because of the complexity of the ecosystem. However, IDS mechanisms are largely significant for different paradigms, as some are considered effective in countering DoS/DDoS attacks (Zero-day-attack). In certain scenarios, IDS mechanisms introduce gateway devices to provide higher processing power if needed.

Security and privacy are considered primary drawbacks, limiting several institutions and organizations to adopt computational offloading technology. As mentioned earlier, these paradigms face different security and privacy threats, but the most outstanding are DoS/DDoS attacks. For instance, Cloud customers can suffer heavily if Cloud services and resources are breached for a moment by attackers. Cloud systems encounter high latency and high costs in communication and data storage. These issues are present because of the centralized nature of the Cloud and its geographical distance from end-devices that produce data. To resolve these shortcomings in the Cloud, Edge Computing was introduced as a Cloud Computing extension.

As identified during the review, Edge provides much less latency than Cloud platform to end-devices; thus, there is a rapid drop in security when migrating from the Cloud platform to the Edge platform due to the Edge network being decentralized (distributed) in nature. Furthermore, observing the migration of data to end-devices from Cloud platform via Edge network, the storage capacity sharply reduces. There is also a rapid decrease in real-time operations as data moves from end-devices via the Edge platform to the Cloud platform. For longer storage needs, a Cloud platform is used. Storage or processing of data from the end-devices occurs in the Edge platform. Despite the emerging of Edge Computing, vulnerabilities and threats still exist, and this, therefore, calls for strict measures with enhanced security and privacy techniques. Fog paradigm was considered to ameliorate Cloud and Edge paradigms.

As with the Edge paradigm, Fog is rendering services (computation, networking, data storage, etc.) closer to the end-devices rather than moving data to the Cloud platform but in a distributed manner. However, the introduction of the Fog paradigm is seen to improve the infrastructural network to match the demands of large data quantity while enhancing the processing strength efficiently. Fog paradigm can improve mobility, complexity in a distribution environment, location identity, real-time response, as well as security and privacy. The fog paradigm does not depend on the Cloud data center but instead relies on end-devices to store and process its data. Broader availability of node access gives some level of flexibility to the applications. Like the Fog paradigm, the Edge paradigm also permits computation handling at the network edge, near where data are generated. What makes the Fog paradigm different from the Edge paradigm is its ability for Fog nodes to interconnect, while the Edge paradigm operates with separate Edge nodes.

Confidentiality, integrity, and availability are information systems’ most significant security and privacy properties. The transfer and storage of data must be confidential, with integrity, and made available. Confidentiality grants data access only to individuals and organizations that own these data. During the transfer of data within the different user layers, the main network, storing and processing data in Cloud, Edge, or Fog paradigm, its access is strongly restricted. Encrypting data is a way of achieving confidentiality. Data correctness and consistency is a model of integrity which avoids information being tampered with or modified. Some mechanisms can be used for verifying sent and received data integrity. Only authorized persons are granted access to available data. Thus, availability determines that data must be available anywhere based on established policies. To attain these expectations, various instruments, patterns, methodologies, and mechanisms such as cryptography, encryption, authentication, and others are deployed to the multiple platforms (layers) when data are being transferred and stored.

Overall, Cloud, Edge, and Fog paradigms exhibit the same view of providing QoS to customers, but they all have a separate set of features that makes them differ from one another, as we have explained in this work. Notably, the Fog paradigm is designated the most effective and reliable system to better handle the security and privacy challenges encountered.

To summarize, even though the Fog paradigm can offer better security and privacy services to end-devices in general, some features of the Fog paradigm, such as decentralization, constraints of resources, homogeneity, and virtualized systems, are vulnerable to security and privacy challenges in comparison to the Cloud paradigm, which is centralized. Due to the absence of standardization regarding countermeasures deployment, highly effective security and privacy mitigation in the Cloud paradigm cannot be implemented straight to the Fog paradigm because of the named features above. Therefore, Fog systems do need innovative countermeasures to address these challenges. Future research should also address new techniques and mechanisms that fit Fog paradigm features and possibly cross-platform countermeasure tools. Hence, they should be suggestions for effective and efficient solutions.

**Review Methodology:** The systematic literature review is based on PRISMA guidelines [10]. The publication date range was set from 2017 to 2021. We used the most popular ICT sector databases for research works, such as IEEE, Web of Science, Science Direct, Springer, and Scopus, while not considering pre-prints, duplicates, and gray literature. Later on, we analyzed the titles, abstracts, and keywords of the various academic publications to figure out specific journal articles and other important papers related to security and privacy in Cloud, Edge, and Fog paradigms. The following search query was formulated for reproducibility: 
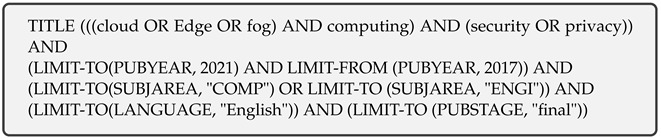

Some exclusion criteria were set to narrow the search outcomes during the first screening stage from the paper’s titles and abstracts:

- Not related to security and privacy in Cloud, Edge, and Fog computing;
- Not in English;
- Works with no technical content;
- Purely review papers;
- Full text not available.

After applying the exclusion criteria, the selected number of publications was lowered from 1390 to 447. Sixty-one duplicates were found and were taken off the list. The headings of the various articles, their abstracts, and important words of the retained 386 papers were screened, and 187 papers were dismissed since they did not match the exclusion criteria. The number of papers left was 199, and their whole content were thoroughly analyzed. After the additional screening, 122 papers were still rejected since they were unrelated to the topic.

## Figures and Tables

**Table 1:** Comparison on different computing paradigms.

Attributes	Cloud Computing	Edge Computing	Fog Computing
Architecture	Centralized	Distributed	Distributed
Expected Task Execution Time ${}^{1}$	High	High-Medium	Low
Provided Services	Universal services	Often uses mobile networks	Vital for a particular domain and distributed
Security	Centralized (guaranteed by the Cloud provider)	Centralized (guaranteed by the Cellular operator)	Mixed (depending on the implementation)
Energy Consumption	High	Low	Varying but higher than for Edge
Identifying location	No	Yes	Yes
Main Providers	Amazon and Google	Cellular network providers	Proprietary
Mobility	Inadequate	Offered with limited support	Supported
Interaction in Real-Time	Available	Available	Available
Latency	High	Low	Varying but higher than for Edge
Bandwidth Cost	High	Low	Low
Storage capacity and Computation	High	Very limited	Varying
Scalability	Average	High	High
Overall usage	Computation distribution for huge data (Google MapReduce), Apps virtualization, Storage of data scalability	Control of traffic, data caching, wearable applications	CCTV surveillance, imaging of subsurface in real-time, IoT, Smart city, Vehicle-to-Vehicle (V2X)

^1^ Importantly, Edge may provide higher results but only for computationally simple tasks (benefiting in terms of communication latency), while Fog would provide higher computational speed maintaining the latency (for, e.g., AR/VR applications). Executions in the Cloud would always provide the worst results as the computational unit is geographically distant from the user, which would naturally require tremendous communication overheads compared to geographically closer locations.

**Table 2:** Attack specifics of paradigms and suggested countermeasures.

Layer	Brief Description	Attack	Specifics of Paradigm/Main Proposed Countermeasures		
			Cloud	Edge	Fog
Application	Data inclined applications faces attacks and if breached, unpermitted access on websites is reached. Malware is of different forms, e.g., Trojan horses and viruses. An illegal software used to access legitimate information. Attacks HTTP [117].	HTTP Flood	Application monitoring is highly recommended. Web Application Firewalls (WAF), Anti-virus, privacy protection management [118].	Filtering mechanisms and intrusion detection systems [26].	HTTP-Redirect scheme [119].
		SQL Injection	SQL injection detection using adaptive deep learning [120].	Modifying circuits to minimize information leakage by adding random noise or delay, implementing a constant execution path code and balancing Hamming weights [121].	SQL injection detection using Elastic-pooling [122].
		Malwares	Use of Antivirus Softwares [118].	Signature-based and behavior-based detection [123].	Mirai botnet detector [119].
Session/Presentation	“It is defined as a pool of virtualized computer resources.” Virtualization offers better usage of hardware assets with an opportunity for additional services avoiding extra costs for infrastructures. Customers are provided with virtual storage [124].	Hyper- visor	Strong configurations, up-to-date Operating System (OS).	Computational Auditing	Robust Authentication scheme.
		Data leakage	Encrypt stored data/use secured transmission medium, e.g., SSL/TLS, Virtual Firewall [125]	Homomorphic Encryption [126].	Isolation of user’s data, Access control strictly based on positions [114].
		VM-Based	Anti-viruses, anti-spyware to monitor illegal events in guest OS [127].	Identity and Authentication scheme such as Identity-Based Encryption (IBE) [126].	Intrusion detection and prevention mechanism use for anomaly detection, behavioral assessment, and machine learning approach in classifying attacks [119].
Transport	“Provides a total end-to-end solution for reliable communications”. The two main protocols are TCP and UDP. The smooth performance in communication strongly depends on TCP/IP between user and server [128].	TCP Flood	Firewalls, SYN Cache [129].	SYN cookies [130].	Integrated Firewalls [131].
		UDP Flood	Graphene design for secure communication [132].	Response rate for UDP packets should be reduced [131].	Response rate for UDP packets same as in Edge, should be reduced [131].
		Session hijacking	AES-GCM symmetric encryption [132].	User light-weight authentication algorithm [130].	Encrypting communication using two-ways or multi-purpose authentication [92].
Network	The routing of data packets across different networks from a source to an end node, is performed by the network layer [133].	DoS attack	Intrusion Detection System (IDS) [134], Access Security	Network Authentication mechanisms	Deploy routing security and observing the behaviour of nodes [135].
		MITM	Data Encryption [118].	Time stamps, encryption algorithm [121].	Use of Authentication schemes [114].
		Spoofing attacks	Identity Authentication [118].	Secure trust schemes [39].	Secured identification and Strong authentication [39].
PHY/MAC	The manner how types of equipment are physically hooked up to a wired or wireless network system and can be sorted for physical addressing with the help of a designated MAC address [136].	Eaves-dropping	Encryption, Cryptography [137]	Data Encryption using asymmetric AES scheme [121].	Protection of identity by use of IBC [138].
		Tampe-ring	Detection of behavioural pattern	Observe manner of behaviour [137].	Multicast authentication as PKI [67].
		Replay attack	Dynamic identity-based authentication model [139].	Authentication mechanisms [140].	Key generation approach [140].

## Abbreviations

The following abbreviations are used in this manuscript:

5G	5th Generation Networks
AES	Advanced Encryption Standard
AP	Access Point
APT	Advanced Persistent Threats
AR	Augmented Reality
BE	Back End
BLE	BLuetooth Low Energy
BS	Base station
CCTV	Closed-circuit television
CSA	Cloud Security Alliance
CSP	Cloud service providers
DDoS	Distributed Denial of Service
DoS	Denial of Service
FE	Front End
FN	Fog Nodes
GCM	Galois/Counter Mode
HTTP	Hypertext Transfer Protocol
LTE	Long Term Evolution
IaaS	Infrastructure as a service
IBC	Identity Based Cryptography
IBE	Identity-Based Encryption
ICT	Information and Communication Technology
IDS	Intrusion Detection System
IoE	Internet of Everything
IoWT	Internet of Wearable Things
MAC	Mediul Access Control
MITM	Man-in-the-Middle Attack
MR	Mixed Reality
NIST	National Institute of 66 Standards and Technology
OS	Operating System
OSI	Open Systems Interconnection model
PaaS	Platform as a Service
PKI	Public Key Infrastructure
PRISMA	Preferred Reporting Items for Systematic Reviews and Meta-Analyses
QoS	Quality of Service
SaaS	Software as a Service
SLA	Service Level Agreement
SQL	Structured Query Language
SSL	Secure Socket Layer
SYN	SYNchronize message
TCP	Transmission Control Protocol
TIP	threat intelligence Platform
TLS	Transport Layer Security
UDP	User Datagram Protocol
V2X	Vehicle-to-Vehicle
VM	Virtual Machines
VR	Virtual Reality
WAF	Web Application Firewalls
Wi-Fi	Wireless Fidelity
